# Process Parameter Modeling and Optimization of Abrasive Water Jet Dressing Fixed-Abrasive Pad Based on Box–Behnken Design

**DOI:** 10.3390/ma15155251

**Published:** 2022-07-29

**Authors:** Zhankui Wang, Shiwei Wang, Yangyang Ding, Yakun Yang, Lijie Ma, Minghua Pang, Jianhai Han, Jianxiu Su

**Affiliations:** 1 Postdoctoral Station, Henan University of Science and Technology, Luoyang 471000, China; 2 Postdoctoral Research Base, Henan Institute of Science and Technology, Xinxiang 453003, China; 3 School of Mechanical and Electrical Engineering, Henan Institute of Science and Technology, Xinxiang 453003, China; wangshiwei_1208@163.com (S.W.); dyy0113163@163.com (Y.D.); yyk2236@163.com (Y.Y.); mlj001@163.com (L.M.); pangminghua909@163.com (M.P.); dlutsu2004@126.com (J.S.); 4 Henan University of Science and Technology, Luoyang 471000, China; jianhaihan@haust.edu.cn

**Keywords:** abrasive water jet, FAP, dressing, response surface method, optimization

## Abstract

Clarifying the influence of the dress process parameters of the abrasive water jet on the dressing effect of fixed-abrasive pads (FAPs) is a prerequisite for online controllable dressing of abrasive water jets. This paper uses three factors and three horizontal response surface methods to explore the influence of jet pressure, abrasive concentration, and nozzle angle on FAP dressing quality. The prediction model of the material removal rate of a FAP machined using three process parameters is established. The influence of pairwise interactions of the three process parameter variables on the dressing effect and the optimal process parameters under each target is analyzed. Finally, the optimal process parameters predicted by the model are verified by experiments. The results show that the best dressing parameters with the MRR of the workpiece as the response value are as follows: jet pressure 3.8 MPa, abrasive concentration 3%, and nozzle angle 73°. The predicted value of the optimal process performance is 464.574 nm/min, and the experimental verification result is 469.136 nm/min; the error between the experimental value and the predicted value is within a reasonable range.

## 1. Introduction

Fixed-abrasive machining technology has the advantages of minor subsurface damage, small surface shape error, and controllable wear particle trajectory, so it is widely used in ultra-precision machining [1,2,3,4]. However, the surface topography of a FAP plays a crucial role in the machining quality of the workpiece [5]. For example, the surface of the FAP will produce a “glazed” phenomenon in the later stage of processing [6], which causes the lapping and polishing efficiency to gradually decrease with the extension of the lapping and polishing times. Therefore, how to reasonably trim a FAP has become a critical technical problem to solve in ultra-precision machining.

Presently, the conventional dressing technology of FAPs can be divided into two categories [7]: self-dressing technology and non-self-dressing technology. Many scholars at home and abroad have carried out related research. Hua Qianfeng et al. [8] analyzed the influence of diamond particle size, arrangement, dressing pressure, time, and rotational speed on the dressing effect of the lapping and polishing pad and discussed the relationship between dressing density and blunt rate. Zhao Wenhong et al. [9] changed the dressing effect of the FAP by adjusting the design, the dressing process parameters, and the processing parameters of the diamond dresser. It is theorized that finishing the FAP will affect the flat effect, polishing rate, and contact area between the lapping and polishing pad and the processing material. Zhu Yongwei’s team [10] studied the effects of pore structure and the composition of lapping and polishing solutions on the self-dressing performance of FAP, optimized the addition ratio of the pore-forming agent in the preparation of the hydrophilic lapping and polishing pad, studied the self-dressing performance of the hydrophilic FAP, and analyzed its self-dressing mechanism. J.Y. Choi et al. [11] prepared several kinds of hydrophilic FAPs with different self-dressing properties by adjusting the ratio of active diluents of hydrophilic resins. They applied them to the grinding test of D2 die steel. It is believed that there is no “glazing” phenomenon in the grinding process of D2 die steel, and the processability of FAP is better. Since the first international conference on water jet cutting technology was held by the British Institute of Fluid Mechanics (BHRA) in 1972, water jet machining technology has been widely used in industrial processing [12,13,14,15,16]. Miyachi et al. [17] used a high-pressure water jet to trim different lapping and polishing pads, which improved the lapping and polishing pads’ dressing effects and reduced the lapping and polishing pads’ dressing bluntness.

To summarize, although existing dressing technology has made some progress, there are still some deficiencies. There are some defects when dressing with diamond dressers, such as the low service life of the lapping and polishing pad, easy scraping of the workpiece [18], low dressing efficiency, and poor finishing quality when dressing with a water jet [19]. In the later stage of lapping and polishing, there is still the specific problem of “glazing” when using a self-repairing FAP [20]. The abrasive water jet machining technology has the characteristics of stable removal function, small removal, low cost, and strong adaptability to the structure and shape of the workpiece, but its application in FAP dressing is still rare. Therefore, a new method of using an abrasive water jet to trim the FAP is being put forward. The results show that the abrasive water jet can effectively trim the lapping and polishing pad. However, the coupling and interaction between the jet system’s process parameters are unclear. For this reason, this paper uses the Box–Behnken experimental design method [21,22,23] to establish a response surface analysis model of three process parameters—jet pressure, abrasive concentration, and nozzle angle—for the material removal rate of polished quartz glass, in order to obtain the influence of process parameters on the dressing effect of the lapping and polishing pad and the pairwise interaction among process parameters. This paper provides theoretical guidance for selecting technological parameters for abrasive water jet dressing and FAPs.

## 2. Experiments

### 2.1. Experimental Materials

**Figure 1:**
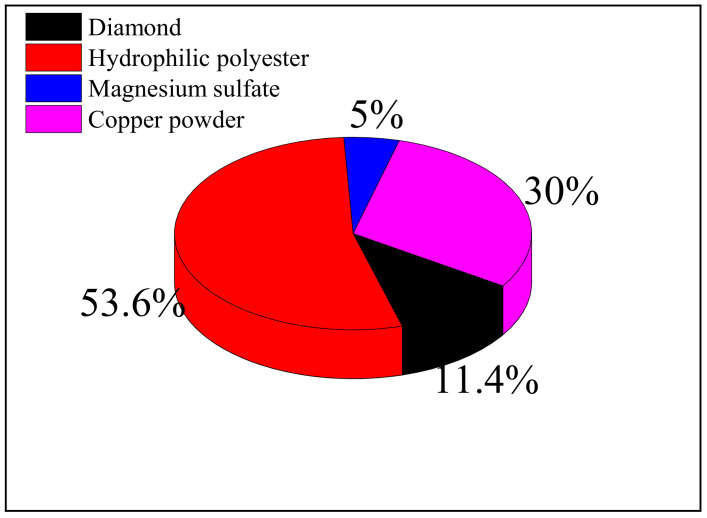
Composition of W7 diamond FAP.

The W7 diamond FAP with 30% copper content was used in the dressing test; its main composition and mass fraction percentage are shown in Figure 1, and the specific preparation process is shown in reference [24]. The abrasive used in the abrasive water jet system is 3.5 μm of brown corundum. The workpiece used in the grinding test was a round quartz glass of φ 25 mm × 3 mm, and its physical and mechanical properties are shown in Table 1. The grinding fluid used in the grinding test was ultrapure, deionized water with an electrical conductivity of 18.24 MΩ.

### 2.2. Experimental Setup

**Figure 2:**
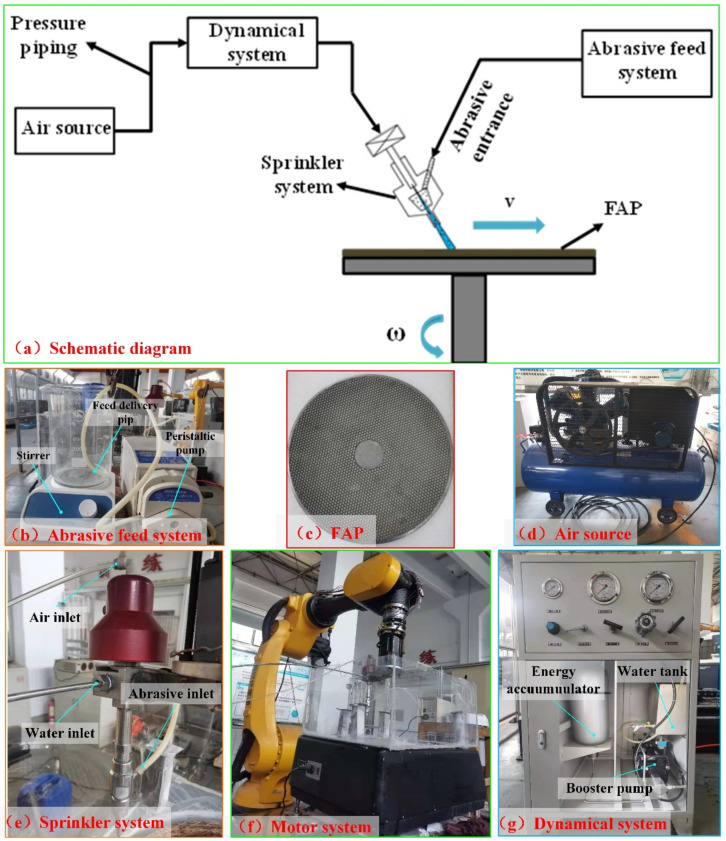
Principle (**a**) and physical diagram of abrasive jet dressing system (**b**–**g**).

The abrasive water jet dressing system used in the dressing test was designed and built by us, and was mainly composed of a power system, an abrasive supply system, a sprinkler system, and a motion control system. Its principle and physical designs are shown in Figure 2. Before each dressing experiment, the marathon grinding experiment was carried out on the FAP, and the grinding time was 150 min. The surface blunt condition of the FAP was dressed with a unified abrasive jet. In order to verify the dressing effect of the FAP after being dressed by the jet dressing system with different factors, the FAP was used to grind the quartz glass on the ZDHP-30 B ultra-precision plane lapping and polishing machine. The process parameters of the grinding test are shown in Table 2. The quartz glass processed before each grinding test was roughed under the same conditions to determine the consistency of the quartz glass surface to be processed.

### 2.3. Experimental Design

The self-built jet system was used to carry out the dressing test, and the method used in this test was the response surface method Box–Behnken design (BBD), the principle of which can be found in reference [25]. The factors examined were jet pressure *X*_1_, abrasive concentration *X*_2_, and sprinkler angle *X*_3_. The selected levels of these three factors were: *X*_1_ 3 MPa, 4 MPa, and 5 MPa; *X*_2_ 3%, 5%, and 7%; and *X*_3_ 60°, 70°, and 80°. The material removal rate of quartz glass, processed for the first time by the FAP with different process parameters, was taken as the response value. The 1 level, 0 level, and −1 levels of each processing parameter variable were represented by Xip2i, Xtun0i, and Xtun1i, respectively. The above three parameters were coded using Equation (1):

(1) $$X_{i}\;=\;\frac{\;X_{i}-X_{0}}{∆i}\;\;\;\;\;i=1,\;2,\;3$$

where $X_{i}$ is the coded value of the variable; $\;x_{i}$ is the actual value of the processing parameter variable; and $x_{0}\;$ is the 0-level actual value of the process parameter variable and the variation range of the true value range. The corresponding table of factor levels and coding values are shown in Table 3, and the other parameters of the jet system are shown in Table 4.

### 2.4. Evaluation Indicators

The dressing effect of the FAP is directly related to the processability of the quartz glass after finishing. That is, the better the dressing effect of the FAP, the better the processability of the quartz glass. Therefore, the material removal rate of the quartz glass processed by the FAP was selected as the quantitative evaluation index of the dressing effect of the FAP. The surface morphology of the FAP before and after dressing and the surface morphology of processed quartz glass were selected as the qualitative evaluation index of the dressing effect of the FAP.

The quality of the quartz glass samples before and after processing was weighed using the SatoriousBSA2245-CW precision electronic balance (0.1 mg), and the material removal rate MRR was calculated via the “weight loss method” (Formula (2)):

(2) $$\mathrm{MRR}=\frac{∆m}{\rho t\pi r^{2}}\times 10^{9}$$

where MRR is the material removal rate, nm/min; ∆m is the mass change in quartz glass, g; ρ is the quartz glass density, and the test sample shows ρ is 2.2 g/${\mathrm{cm}}^{3}$; t is the processing time, min; and r is the experimental quartz wafer radius, mm.

A Leica DVM6 ultra-depth-of-field microscope was used to detect the surface morphology of the FAPs before and after finishing, without factor level, and a ContourGT-X3/X8 white light interferometer was used to detect the surface morphology of the quartz glass before and after finishing, without factor level.

## 3. Results and Discussion

### 3.1. Experimental Results and Regression Equations

Before dressing, the material removal rate of the FAP-machined workpiece was significantly reduced after being blunted by the marathon experiment, and the material removal rate of its lapping quartz glass was about 150 nm/min. The material removal rate of quartz glass machined by the FAP after dressing with different process parameters improved, and the material removal rate of quartz glass processed for the first time by the FAP after dressing with different process parameters was used as the evaluation index; the test results are shown in Table 5. Through parameter conversion, the free variables of jet pressure *X*_1_, abrasive concentration *X*_2_, nozzle angle *X*_3_, and material removal rate of quartz glass were converted into matrix form, and the regression coefficient was obtained using the least square method. The multiple regression equations of jet pressure *X*_1_, abrasive concentration *X*_2_, nozzle angle *X*_3_, and the material removal rate of quartz glass was obtained, as shown in Equation(3).

(3) $$\mathrm{MRR}=380.1-27.6X_{1}-59.4X_{2}-1.2X_{3}-116.2X_{1}{}^{2}+18.1X_{2}{}^{2}-49.8X_{3}{}^{2}+\;40.9X_{1}X_{2}-12.7X_{1}X_{3}-6.6X_{2}X_{3}$$

### 3.2. Material Removal Rate Model and Its Analysis

#### 3.2.1. ANOVA and Significance Testing of the Material Removal Rate Model

This section presents the significance test and variance analysis using Formula (3). Using the significance of the variance and correlation coefficient judgment in Formula (3), the results of the material removal rate regression model variance analysis of the obtained quartz glass are shown in Table 6. The mean square sum AdjSS represents the change in the data. The mean square value AdjMS is the quotient obtained by dividing the square sum of the mean deviation by the corresponding degrees of freedom. The F value is the mean square ratio, which is used to test the significance of the factor. The *p*-value is the significance level of the factor. It can be seen from Table 6 that the quadratic misfit = 0.133 > 0.05, the correlation coefficient R^2^ = 0.9570, and the adjustment determination coefficient $R_{Adj}^{2}$ = 0.8796, indicating that the regression model of the material removal rate of quartz glass can explain the response value of 87.96%. Therefore, the regression model of material removal rate obtained in this paper fits well and can predict the material removal rate of quartz glass by the FAP after jet system dressing. The results of Table 6 show that *X*_2_ and *X*_1_^2^ in Equation (2) were highly significant at the significance level of *p* < 0.01; *X*_1_*, X*_3_^2^, and *X*_1_ ∗ *X*_2_ were significant at the *p* < 0.05 level, indicating that the relevant factors and their interactions had a significant influence on the material removal rate of quartz glass.

#### 3.2.2. Composite Analysis of the Main Factors Affecting the Material Removal Rate of Quartz Glass

**Figure 3:**
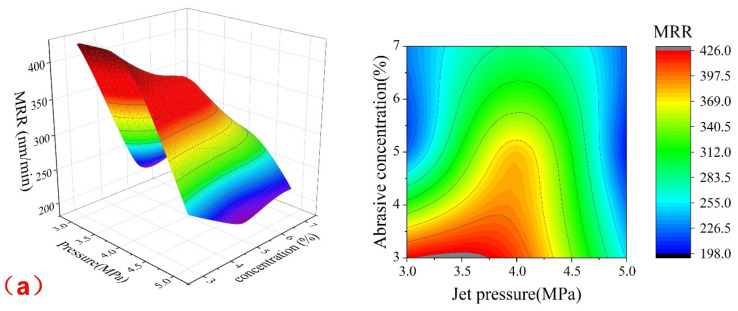

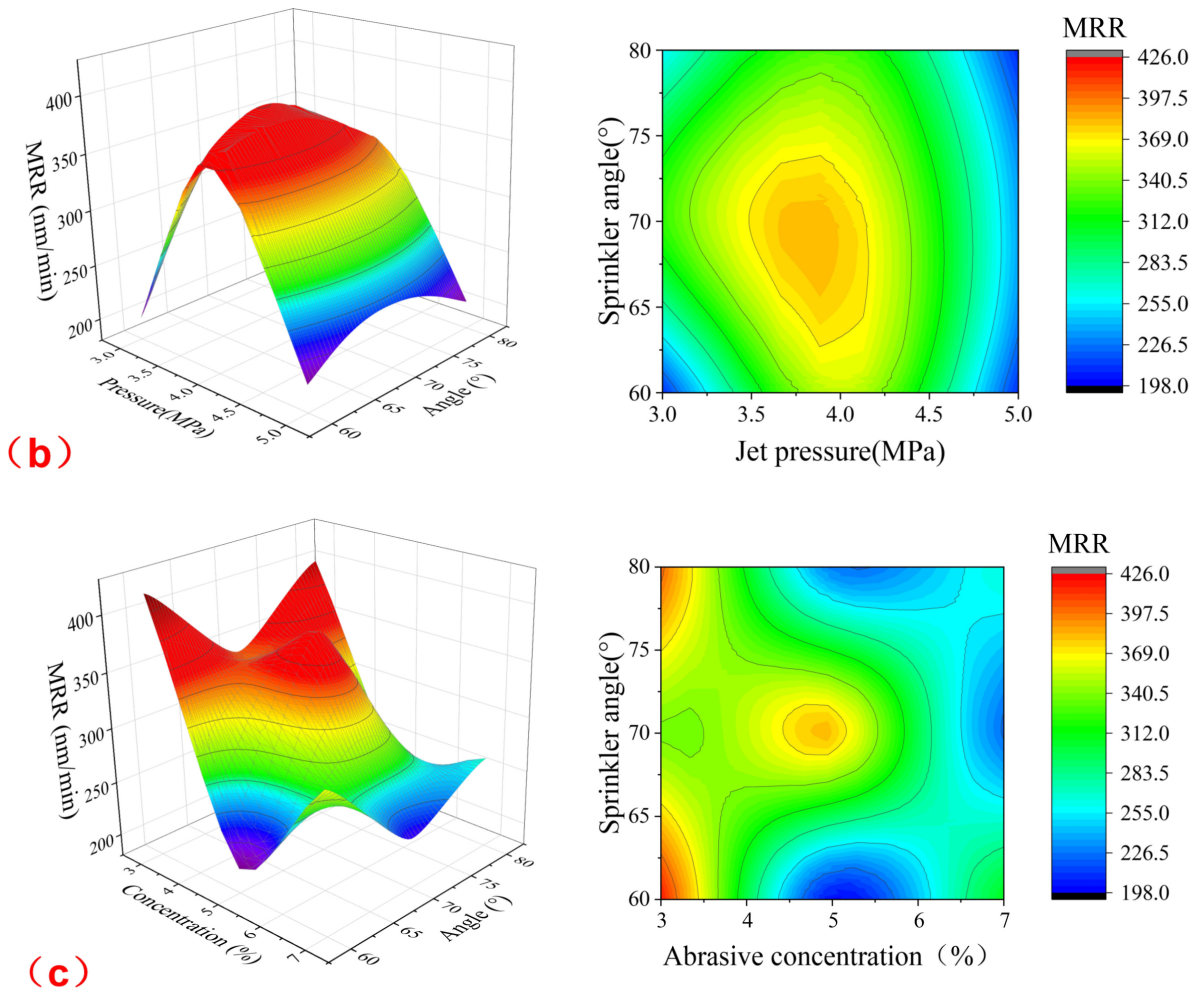
Response surface and contour plot of material removal rate and various factors: (**a**) Response surface and contour plot of material removal rate and jet pressure and abrasive concentration; (**b**) response surface and contour plot of material removal rate to jet pressure and nozzle angle; (**c**) response surface and contour plot of material removal rate to nozzle angle and abrasive concentration.

According to the experimental results in Table 5 and the regression analysis Equation (3), the response surface and contour map of the pairwise compound effect of the leading experimental parameters on the material removal rate of the quartz glass could be obtained using the control variates, as shown in Figure 3.

Figure 3a shows the combined effect of jet pressure and abrasive concentration on the removal rate of quartz glass. Combined with Table 5 and the regression analysis Equation (3), when the nozzle angle is constant, the material removal rate of quartz glass decreases with the increase in the product of jet pressure and abrasive concentration. This is because the lower abrasive concentration has a poor dressing effect on the FAP, but with the gradual increase in pressure, the energy obtained by the abrasive increases, the amount of material removed from the surface of the FAP increases, and the abrasive particles on the surface are exposed. At this time, the dressing effect of the FAP is better. That is, the material removal rate of quartz glass increases gradually. However, when the pressure exceeds 4 MPa, the interference between abrasives is enhanced, and the mixing effect of abrasive and high-pressure water in the mixing cavity is poor. At this time, the dressing effect of the FAP is poor, which reduces the material removal rate of the quartz glass. When the concentration of abrasives increases gradually, there is interference between abrasive particles. With the increase in pressure, the interference between abrasives increases, the energy obtained by abrasives is lower, and the dressing effect of the FAP is poor. The exposure effect of abrasive particles on the surface is poor. That is, the material removal rate of the quartz glass is reduced. Therefore, the interaction between the jet pressure and the abrasive concentration results from the comprehensive action of the above factors. When the jet pressure is 3–4.3 MPa, and the abrasive concentration is 3–3.5%, the dressing effect of the FAP is the best. The material removal rate of quartz glass processed by the dressing FAP is the highest.

Figure 3b shows the combined effect of jet pressure and nozzle angle on the removal rate of quartz glass. Combined with Table 5 and the regression analysis using Equation (3), when the abrasive concentration is constant, the material removal rate of quartz glass increases at first, and then decreases with the increase in the production of jet pressure and nozzle angle. This is because when the angle of the nozzle is slight, the vertical component of the abrasive jet is minor, and the amount of material removed from the surface of the lapping and polishing pad is smaller, so the dressing effect is poor. However, with the increased jet pressure, the vertical component of the abrasive jet increases, the amount of material removed from the surface of the FAP increases, and the dressing effect is better. When the pressure exceeds 4 MPa, the vertical component of the jet is too large, resulting in excessive removal of the surface of the FAP, so the dressing effect is poor. When the nozzle angle increases, although the vertical component of the jet increases with the increase in the nozzle angle, the lower pressure makes the energy of the abrasive jet lower, so the vertical component of the jet does not increase obviously, and the dressing effect is poor; with the pressure increases, the vertical component of the jet increases, and the dressing effect is the best. However, the pressure and nozzle angle continuing to increase will cause excessive removal of the surface of the FAP, so the dressing effect is poor. As a result, the material removal rate of quartz glass processed by the FAP is reduced. Therefore, the interaction between the nozzle angle and the jet pressure result from the comprehensive action of the above factors, and when the jet pressure is 3.5–4.25 MPa and the nozzle angle is 62–73°, the dressing effect of the lapping and polishing pad is the best. The material removal rate of quartz glass processed by the dressing pad is also the highest.

Figure 3c shows the combined effect of abrasive concentration and nozzle angle on the removal rate of quartz glass. Combined with Table 5 and the regression analysis of Equation (3), when the jet pressure is constant, the material removal rate of quartz glass decreases at first, then increases, and then decreases with the increase in the product of the abrasive concentration and nozzle angle. This is because when the jet pressure is constant, the abrasive concentration and nozzle angle increases the interference between abrasive particles, significantly affecting the dressing effect. On the contrary, when the abrasive concentration is low, the nozzle angle is adjusted, and a better dressing effect is achieved. Therefore, the dressing effect of the FAP is the best when the abrasive concentration is 3–5.25%, the nozzle angle is 60–80°, and the material removal rate of quartz glass processed by the dressing pad is the highest.

### 3.3. The Surface Morphology of FAP and Quartz Glass

**Figure 4:**
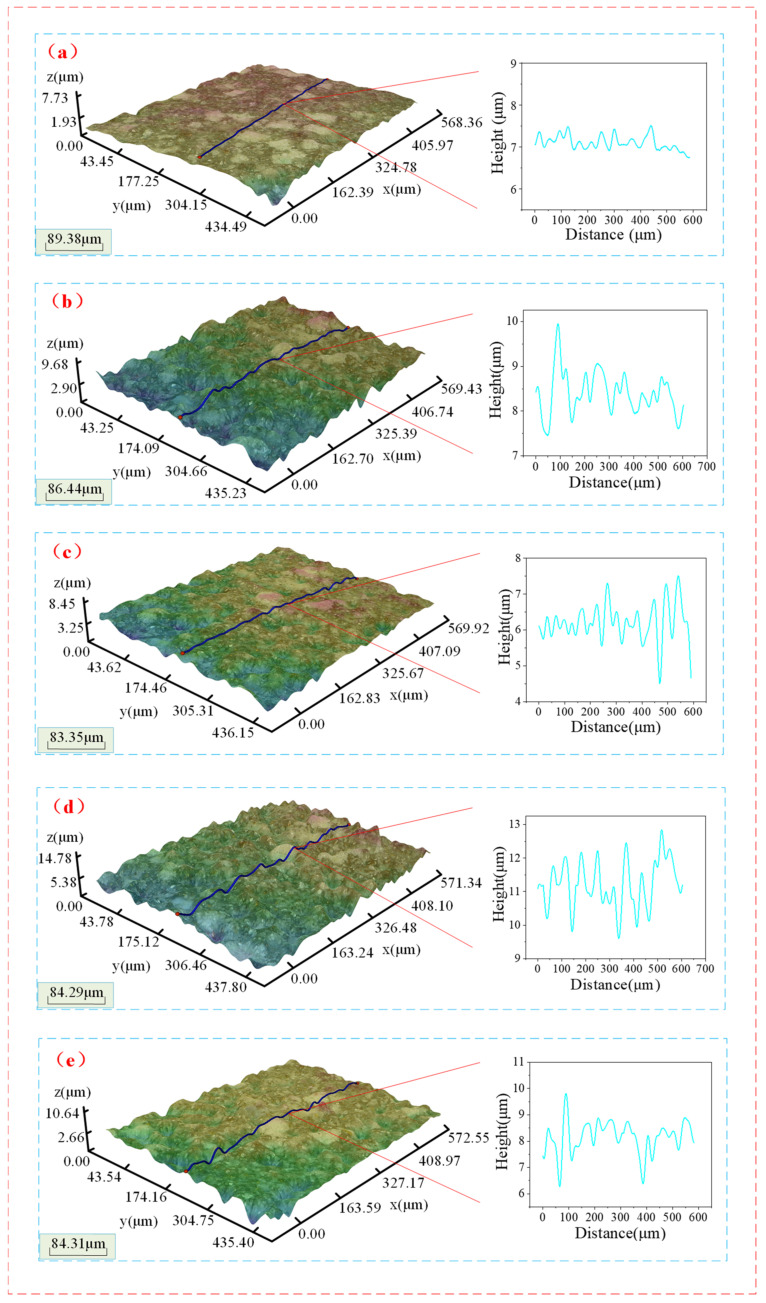
The surface morphology of some samples after dressing FAP by abrasive jet system: (**a**) the surface of the blunt FAP; (**b**) Experiment No. 1 on the surface of FAP; (**c**) Experiment No. 4 on the surface of FAP; (**d**) Experiment No. 8 on the surface of FAP; (**e**) Experiment No. 15 on the surface of FAP.

**Figure 5:**
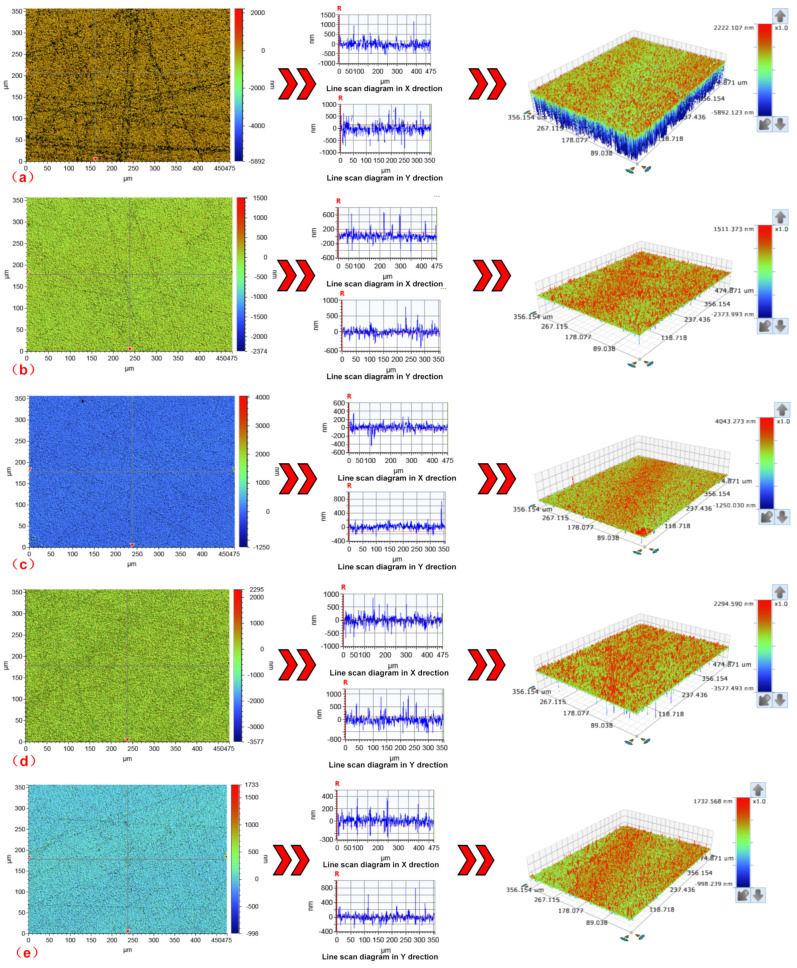
The surface morphology of the first processing of quartz glass by FAP using different process parameters: (**a**) the surface morphology of the quartz glass processed by the blunt FAP; (**b**) the surface morphology of experiment No. 1 quartz glass; (**c**) the surface morphology of experiment No. 4 quartz glass; (**d**) the surface morphology of experiment No. 8 quartz glass; (**e**) the surface morphology of experiment No. 15 quartz glass.

Figure 4 shows the surface morphology of some samples after dressing the FAP using the abrasive jet system. It can be seen from Figure 4 that, compared with the surface of the blunt FAP (Figure 4a), the surface of the FAP dressed by the abrasive jet system shows different degrees of concave and convex peaks, the blunt particles on the surface of the FAP are exposed, and its surface roughness is increased. The processing performance of the FAP has been improved. Figure 5 shows the surface morphology of quartz glass machined by the corresponding jet system in Figure 4 after dressing the FAP. The blunt FAP processes quartz glass; there are many rough scratches on the surface (Figure 5a), and the surface quality is poor. The surface quality of quartz glass processed by the FAP that is processed by a jet system with different process parameters is obviously improved. There are only small scratches on the surface.

### 3.4. Parameter Optimization and Validation

**Figure 6:**
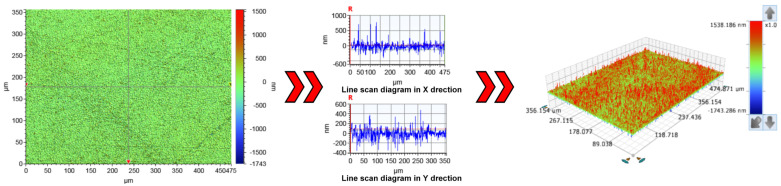
Surface morphology of processed quartz glass after dressing FAP.

The optimum process parameter ranges of jet pressure, abrasive concentration, and nozzle angle analyzed in Figure 3 are converted into a coding value through Formula (1). The converted coding value is brought into the regression Equation (3). Combined with the specific conditions of the test equipment, the optimal process parameters for the maximum material removal rate of quartz glass are as follows: jet pressure 3.8 MPa, abrasive concentration 3%, and nozzle angle 73°. Currently, the predicted material removal rate is 464.574 nm/min. In order to test the reliability of the response surface method, it is necessary to verify the optimal process parameters obtained via the model optimization and the actual material removal rate of quartz glass after lapping and polishing under the optimal process parameters of 469.136 nm/min. Comparing the above two data sets shows that the predicted results are very close to the actual experimental results, except for a difference of 4.562 nm/min. It shows that the optimization result of the dressing effect of the abrasive water jet dressing pad by the response surface method is very reliable. The regression equation obtained by the response surface method has a good prediction ability. The surface morphology of the quartz glass processed by the dressed FAP after finishing is shown in Figure 6. The surface has no rough scratches, is smooth, and has good processing quality.

## 4. Conclusions

Through analysis of the response surface and contour map, the range of the best process parameters could be analyzed accurately, and the regression model could be used to optimize the process parameters. The following conclusions were drawn:

1. The response surface and contour map can accurately analyze the jet system’s best range of process parameters, and when combined with the regression model, can optimize the process parameters of the jet system.
2. After optimization, the best process parameters corresponding to quartz glass’s maximum material removal rate are jet pressure 3.8 MPa, abrasive concentration 3%, and nozzle angle 73°. That is, these process parameters are the best FAP dressing process parameters.
3. By substituting the predicted optimal process parameters into the regression model, the predicted material removal rate of quartz glass is 464.574 nm/min. The experimental results show that the actual material removal rate of quartz glass is 469.136 nm/min. The predicted results are very close to the actual experimental results, except for a difference of 4.562 nm/min.
4. The response surface model established in this paper has high accuracy and can be used to predict the results of an abrasive water jet dressing FAP.

## Figures and Tables

**Table 1:** Physical and mechanical properties of quartz glass.

Properties	Value
Density	2.20–2.21 g/cm^3^
Mohs hardness	6.0–7.0
Elasticity modulus	77.8 GPa
Poisson ratio	0.14–0.17
Breaking tenacity	0.75–0.80 MPa·m^1/2^

**Table 2:** Lapping experimental process parameters.

Lapping Pressure	Types of Abrasive Fluids	Abrasive Fluid Flow Rate	Lapping Speed	Lapping Time
27 KPa	Deionized water	50 mL/min	100 r/min	30 min

**Table 3:** Factor level and coded value correspondence table.

Factor Encoded Value	Jet Pressure A/MPa	Abrasive Concentration B/%	Sprinkler Angle C/°
Up level (+1)	5	7	80
Lower level (−1)	3	3	60
Zero level (0)	4	5	70
Change radius ∆i	1	2	10

**Table 4:** Process parameters of the jet system.

Abrasive Particle Size	Abrasive Flow Rate	FAP Rotate Speed	Traverse Velocity	Dressing Time	Abrasive Class
W3.5	102 g/min	110 r/min	2.5 mm/s	5 min	Brown corundum

**Table 5:** Test results.

	Factors	Jet Pressure A/MPa	Abrasive Concentration B/%	Sprinkler Angle C/°	MRR/Nm/Min
Test Number					
1		−1	−1	0	425.15
2		1	1	0	220.68
3		0	1	−1	308.64
4		1	−1	0	253.09
5		−1	1	0	229.17
6		1	0	−1	203.70
7		0	0	0	364.97
8		−1	0	−1	198.30
9		0	−1	−1	418.98
10		0	−1	1	401.23
11		0	1	1	264.66
12		0	0	0	393.52
13		−1	0	1	250.00
14		1	0	1	204.48
15		0	0	0	381.94

**Table 6:** The results of the material removal rate regression model variance analysis.

Source	Degrees of Freedom	Adj SS	Adj MS	F Value	p Value	Significance
Model	9	101,251	11,250.1	12.36	0.006	**
*X_1_*	1	6087	6087.4	6.69	0.049	*
*X_2_*	1	28,240	28,239.8	31.03	0.003	**
*X_3_*	1	11	10.7	0.01	0.918	
Square	3	59,403	19,800.9	21.76	0.003	**
*X*_1_^2^	1	49,847	49,847.3	54.77	0.001	**
X_2_^2^	1	1205	1205.4	1.32	0.302	
*X*_3_^2^	1	9169	9169.1	10.07	0.025	*
Two-way interaction	3	7510	2503.3	2.75	0.152	
*X*_1_ ∗ *X*_2_	1	6690	6689.6	7.35	0.042	*
*X*_1_ ∗ *X*_3_	1	648	648.4	0.71	0.437	
*X*_2_ ∗ *X*_3_	1	172	172.1	0.19	0.682	
Errors	5	4551	910.1			
Missing fit	3	4138	1379.4	6.69	0.133	
Pure error	2	412	206.2			
Total	14	105,801				
$R^{2}\;$$=0.9570\;\;\;\;\;\;R_{Adj}^{2}\;$= 0.8796						

Note: When the *p* value < 0.01, it indicates a very significant level, which is expressed as **; when the *p* value < 0.05, it indicates a significant level, expressed as *.

## Data Availability

All data generated or analyzed during this study are included in this manuscript.
